# Enhanced Hydroxide Conductivity and Dimensional Stability with Blended Membranes Containing Hyperbranched PAES/Linear PPO as Anion Exchange Membranes

**DOI:** 10.3390/polym12123011

**Published:** 2020-12-16

**Authors:** Sang Hee Kim, Kyu Ha Lee, Ji Young Chu, Ae Rhan Kim, Dong Jin Yoo

**Affiliations:** 1 Department of Energy Storage/Conversion Engineering, Hydrogen and Fuel Cell Research Center, Jeonbuk National University, Jeonju 54896, Jeollabuk-do, Korea; tkdgml3257@naver.com (S.H.K.); canutech@hanmail.net (A.R.K.); 2 Department of Life Sciences, Jeonbuk National University, Jeonju 54896, Jeollabuk-do, Korea; ebbuneg@hanmail.net (K.H.L.); carumiss@naver.com (J.Y.C.)

**Keywords:** anion exchange membrane, hyperbranched polymer, alkaline fuel cell, blended membranes, anion conductivity, dimensional stability

## Abstract

A series of novel blended anion exchange membranes (AEMs) were prepared with hyperbranched brominated poly(arylene ether sulfone) (Br-HB-PAES) and linear chloromethylated poly(phenylene oxide) (CM-PPO). The as-prepared blended membranes were fabricated with different weight ratios of Br-HB-PAES to CM-PPO, and the quaternization reaction for introducing the ionic functional group was performed by triethylamine. The Q-PAES/PPO-XY (quaternized-PAES/PPO-XY) blended membranes promoted the ion channel formation as the strong hydrogen bonds interconnecting the two polymers were maintained, and showed an improved hydroxide conductivity with excellent thermal behavior. In particular, the Q-PAES/PPO-55 membrane showed a very high hydroxide ion conductivity (90.9 mS cm^−1^) compared to the pristine Q-HB-PAES membrane (32.8 mS cm^−1^), a result supported by the morphology of the membrane as determined by the AFM analysis. In addition, the rigid hyperbranched structure showed a suppressed swelling ratio of 17.9–24.9% despite an excessive water uptake of 33.2–50.3% at 90 °C, and demonstrated a remarkable alkaline stability under 2.0 M KOH conditions over 1000 h.

## 1. Introduction

Fuel cells (FCs) are a promising energy conversion device, that can directly convert chemical energy into electrical energy [1,2,3]. Among the various existing FCs, cation exchange membrane fuel cells (CEMFCs) have received particular attention due to their many advantages, such as high power density and superior efficiency [4,5]. Despite these advantages, the commercialization of the CEMFC has been difficult due to the high system costs associated with the use of precious metal platinum catalysts and fluorinated membranes [6]. On the other hand, anion exchange membrane fuel cells (AEMFCs) are capable of reducing system costs by using less valuable metal catalysts (Co and Ni) [7] and have been studied recently due to their advantages, including fast oxygen reduction reactions (ORR) at the cathode, low fuel crossover, and easy water management. To advance AEMFCs, the development of an anion exchange membrane (AEM) with high ionic conductivity and physicochemical stability is essential [8]. However, the AEMs that have been studied thus far yield an insufficient electrochemical performance due to the inherently lower mobility of OH^−^ ions compared to H^+^ ions and unstable dimensional stability due to the excessive swelling of the AEM [9,10,11,12]. Therefore, many researchers have studied AEM with high ion exchange capacity (IEC) value as a method of improving ionic conductivity. However, a high IEC value causes excessive dimensional change in the membrane along with a high water uptake, which leads to separation of the membrane electrode assembly (MEA) during the manufacture and deterioration of fuel cell durability from the wet-dry repetition [13,14]. As mentioned above, various studies have achieved excellent dimensional stability along with high IEC values such as blended membranes [15,16,17,18]; inorganic/organic composite membranes [6,19,20]; and the backbone design of comb-shaped [21,22,23], branching [24,25,26], and cross-linking [27,28].

Novel hyperbranched anion exchange membranes (HBAEMs) are promising backbone structures for AEMs that have been reported to possess improved dimensional stability, alkaline stability, and clear micro-phase separation of hydrophilic/hydrophobic domains, which result in excellent ionic conductivity and high solubility enabling facile membrane fabrication and reprocessing compared to other polymers [29,30,31,32]. Ge et al. [33] reported hyperbranched poly-4-(chloromethyl) styrene (HB-PVBC) AEMs with superior dimensional stability and an ionic conductivity of 123.01 mS cm^−1^ at 80 °C, which results from the well-separated hydrophilic/hydrophobic microphases and the low swelling ratio of HB-PVBC due to its hyperbranched structure.

In addition, a promising strategy for the development of AEMs with excellent ionic conductivity, mechanical properties, and thermal stability is to blend polymers to achieve a balance of physicochemical stability and electrochemical performance [34]. For example, Kim et al. [35,36] reported that miscible blended membranes comprised of sulfonated-fluorinated, hydrophilic-hydrophobic copolymer and sulfonated poly(ether ketone) demonstrated good phase-separated morphology and superior ionic conductivity.

Recently, the aromatic polymer, poly(phenylene oxide) (PPO) has been used in many applications due to its good thermal stability, excellent mechanical strength, improved conductivity via the introduction of functional groups, and facile synthesis [37]. Xu et al. [38] reported that the development of a series of PPO-based AEMs with ethylene oxide spacers demonstrated a very good peak power density (437 mW cm^−2^).

In accordance with these studies, we designed a blended membrane of hyperbranched poly(arylene ether sulfone) (HB-PAES) and the linear polymer poly(phenylene oxide) (PPO) as a novel strategy. This strategy aims to compensate for each polymer’s drawbacks [39]. In this study, bromination and chloromethylation reactions were performed to introduce quaternary functional groups into HBPAES based on the hyperbranched structure and PPO polymer, respectively, and the structure of the prepared polymers was confirmed through ^1^H NMR, FT-IR, and GPC analyses. Subsequently, a series of blended polymers were fabricated by adjusting the mixing ratio of HBPAES to PPO (3:7, 4:6, 5:5, 6:4, and 7:3) to investigate, the physicochemical stability and electrochemical properties affected by the mixing ratio.

## 2. Materials and Methods

### 2.1. Materials

1,1,1-Tris(4-hydroxyphenyl)ethane (THPE) and 2,2-bis(4-hydroxy-3-methylphenyl)propane (BHMP) were purchased from Tokyo Chemical Industries (Tokyo, Japan). Bis-(4-chlorophentyl)sulfone (BPS), N-bromosuccinimide (NBS), anhydrous toluene, anhydrous *N*,*N*-dimethylacetamide (DMAc), poly(2,6-dimethyl-1,4-phenylene oxide) (PPO), and triethylamine (TEA) were obtained from Sigma-Aldrich (Seoul, Korea). Potassium carbonate (K_2_CO_3_), benzoyl peroxide (BPO), 1,1,2,2-tetrachloroethane (TCE), ethanol, methanol, and acetone were purchased from Daejung Chemicals and Metals company (Seoul, Korea).

### 2.2. Synthesis of the Hyperbranched Hydrophobic Oligomer

**Scheme 1:**
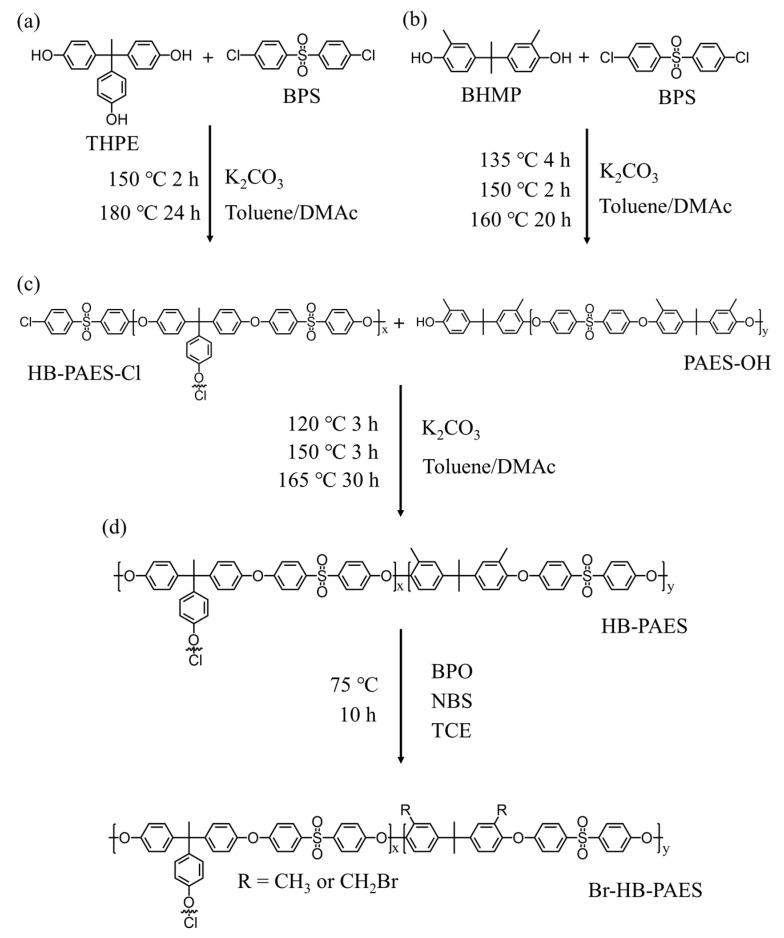
Synthesis of (**a**) HB-PAES-Cl, (**b**) PAES-OH, (**c**) HB-PAES, and (**d**) Br-HB-PAES.

The hyperbranched Cl-terminated poly(arylene ether sulfone) hydrophobic oligomer (HB-PAES-Cl) was synthesized via the aromatic nucleophilic substitution reaction (Scheme 1a). The polymerization of HB-PAES-Cl was carried out in THPE (1.25 g, 4.08 mmol), BPS (2.58 g, 8.98 mmol), K_2_CO_3_ (1.69 g, 12.24 mmol), DMAc (30.0 mL), and toluene (15.0 mL) in a round-bottomed flask equipped with a Dean-Stark trap and a reflux condenser under nitrogen gas. The mixture was heated to 150 °C under stirring, and its temperature was maintained at 150 °C for 5 h to remove azeotropes (toluene and water). Subsequently, polymerization was carried out at 180 °C for 24 h. Afterward, the mixture was cooled at room temperature, and the viscous solution was poured into a mixture of methanol, acetone, and distilled water (DI water) (*v*/*v*/*v*, 7/1/1). The precipitated powder was collected by filtration, and the white fiber product was dried in a vacuum oven at 70 °C for 24 h (yield: 92%). The chemical structure of the as-synthesized HB-PAES-Cl was characterized using ^1^H NMR, as shown in Figure S1a (600 MHz, DMSO-d_6_: 2.17–2.00 ppm (H_n_), 7.16–6.84 ppm (H_h_, H_h′_, H_l_, H_m_), 7.69–7.54 ppm (H_k_), 7.97–7.75 ppm (H_i′_, H_j_, H_j′_), and GPC (*M_n_* = 7 kDa, *M_w_* = 22 kDa).

### 2.3. Synthesis of the Hydrophilic Oligomer

The OH-terminated poly(arylene ether sulfone) hydrophilic oligomer (PAES-OH) was synthesized via the aromatic nucleophilic substitution reaction, in a process similar to that used for HB-PAES-Cl [40]. As shown in Scheme 1b, the polymerization of PAES-OH was performed with BHMP (1.96 g, 7.66 mmol), BPS (2.00 g, 6.96 mmol), K_2_CO_3_ (2.12 g, 15.32 mmol), DMAc (20.0 mL), and toluene (15.0 mL) in a round-bottomed flask equipped with a Dean-Stark trap and a reflux condenser under nitrogen gas. The reaction mixture was heated to 135 °C under stirring, and its temperature was maintained at 150 °C for 2 h to remove azeotropes (toluene and water). Afterward, polymerization was carried out at 160 °C for 20 h. Subsequently, the mixture was cooled at room temperature, and the viscous solution was poured into a mixture of methanol, acetone, and DI water (*v*/*v*/*v*, 7/1/1). Finally, the precipitated powder was collected by filtration, and the light brown product was dried in a vacuum oven at 70 °C for 15 h (yield: 94%). The chemical structure of the as-synthesized HTHP-OH was characterized using ^1^H NMR, as shown in Figure S1b (600 MHz, DMSO-d_6_: 1.66–1.39 ppm (H_g_), 2.07–1.81 ppm (H_f_), 7.07–6.63 ppm (H_a_, H_b_, H_d_), 7.26–7.12 ppm (H_c_), 7.87–7.69 ppm (H_e_), and GPC (*M_n_* = 10 kDa, *M*_w_ *=* 22 kDa).

### 2.4. Synthesis of Hyperbranched Poly(Arylene Ether Sulfone) Block Copolymer

The hyperbranched poly(arylene ether sulfone) (HB-PAES) was prepared via the aromatic nucleophilic substitution reaction (Scheme 1c). The polymerization of HB-PAES was conducted with HB-PAES-Cl (2.00 g, 0.19 mmol), PAES-OH (2.14 g, 0.19 mmol), K_2_CO_3_ (0.05 g, 0.38 mmol), DMAc (25.0 mL), and toluene (12.0 mL) in a round-bottomed flask equipped with a Dean-Stark trap and reflux condenser under nitrogen flow. The reaction mixture was heated at 120 °C under stirring, and the mixture temperature was maintained at 150 °C for 3 h to remove azeotrope (toluene and water). Afterward, the polymerization was carried out at 165 °C for 30 h. Subsequently, the mixture was cooled to room temperature, and the viscous solution was poured into a mixture of methanol, acetone, and DI water (*v*/*v*/*v*, 7/1/1). Finally, the precipitated powder was collected by filtration, and the light brown product was dried in a vacuum oven at 70 °C for 15 h (yield: 90%). The chemical structure of the as-synthesized HB-PAES was characterized using ^1^H NMR, as shown in Figure S1c (600 MHz, DMSO-d_6_: 1.65–1.46 ppm (H_g_), 2.15–1.80 ppm (H_f_, H_n_), 7.23–6.70 ppm (H_a_, H_b_, H_d_, H_h_, H_h′_, H_l_, H_m_), 7.26–7.25 ppm (H_c_), 7.55–7.54 ppm (H_k_), 7.95–7.55 ppm (H_e_, H_i_, H_i′_, H_j_), and GPC (*M_n_* = 97 kDa, *M_w_* = 153 kDa).

### 2.5. Synthesis of Brominated HB-PAES (Br-HB-PAES)

The brominated hyperbranched poly(arylene ether sulfone) (Br-HB-PAES) was synthesized via the Friedel-Crafts alkylation (Scheme 1d) [41,42,43]. The bromination reaction of Br-HB-PAES was performed with HB-PAES (3.00 g, 0.14 mmol), TCE (30.0 mL), and NBS (4.49 g, 6.30 mmol) as the bromination reagent, with BPO (0.61 g, 0.63 mmol) as the initiator in a two-neck round bottom flask equipped with a reflux condenser under nitrogen flow. The reaction solution was heated at 40 °C under stirring, and the bromination reaction was carried out at 75 °C for 10 h. Afterward, the mixture was cooled at room temperature, and the yellow product was poured into methanol (700 mL). Finally, the precipitated powder was collected by filtration, and the yellowish product was dried in a vacuum oven at 70 °C for 15 h (yield: 95%). The chemical structure of the as-synthesized Br-HB-PAES was characterized using ^1^H NMR (600 MHz, DMSO-d_6_: 1.65–1.46 ppm, 2.15–1.80 ppm, 4.60–4.49 ppm, 7.23–6.70 ppm, 7.26–7.25 ppm, 7.55–7.54 ppm, 7.95–7.55 ppm).

### 2.6. Synthesis of Chloromethylated PPO (CM-PPO)

As shown in Scheme S1, for chloromethylation, the CM-PPO copolymer was synthesized via the Friedel-Crafts alkylation [6,41]. The PPO polymer (3.00 g, 0.10 mmol) was dissolved in TCE (15.0 mL) at 30 °C in a two-neck round-bottomed flask equipped with a reflux condenser under nitrogen. Then, chloromethyl ethyl ether (CMME) (7.0 mL) and ZnCl_2_ (0.20 g) as a catalyst dispersed in tetrahydrofuran (THF) (2.0 mL) were added drop-wise to the reaction mixture. Subsequently, the mixture was reacted at 50 °C for 7 days. After cooling, the reaction mixture of chloromethylated PPO (CM-PPO) was precipitated in methanol. Finally, the precipitated powder was collected by filtration, and the white powder was dried in an oven at 60 °C for 24 h. The degree of chloromethylation (DC) was confirmed by ^1^H NMR (600 MHz, DMSO-d_6_: 2.27–1.89 ppm, 4.99–4.61 ppm, 6.14–5.99 ppm, 6.55–6.44 ppm).

### 2.7. Fabrication of the BC-PAES/PPO-XY Blended Membranes

The BC-PAES/PPO-XY (where X and Y represent the weight ratios of Br-HB-PAES and CM-PPO, respectively) blended membranes were prepared by the direct solution casting using different weight ratios of Br-HB-PAES to CM-PPO, as shown in Table 1. For example, the BC-PAES/PPO-55 blended membrane was fabricated with Br-HB-PAES (0.25 g), CM-PPO (0.25 g), and TCE (12.0 mL), and the solution was stirred at 50 °C for 10 h. The solution was cast onto a clean flat glass plate, and dried in a vacuum oven at 70 °C for 13 h. The BC-PAES/PPO-55 blended membrane was peeled off the glass plate using DI water. The fabricated blended membranes were flexible, transparent, and had an average thickness of 30–50 μm.

### 2.8. Quaternization of BC-PAES/PPO-XY Membranes

Quaternized BC-PAES/PPO-XY (Q-PAES/PPO-XY) blended membranes were prepared using triethylamine (TEA) as a tertiary amine for the quaternization reaction. Briefly, the as-prepared membranes were immersed in a 25 wt% triethylamine solution at 50 °C for 12 h to exchange Cl or Br groups for quaternary ammonium groups. Afterward, the quaternized samples were soaked in a 1.0 M KOH solution for 48 h to convert Br^−^ or Cl^−^ forms to OH^−^ forms, and the quaternized membranes were kept in DI water.

## 3. Characterizations

The proton nuclear magnetic resonance (^1^H NMR) spectroscopy was utilized to analyze the chemical structure of the polymers using the JNM-ECA 600 instruments (Bruker installed at the Center for University Wide Research Facilities (CURF) in Jeonbuk National University (JBNU), Jeonju, Korea. The number average molecular weight, weight average molecular weight, and polydispersity index (PDI) of the as-prepared polymers were estimated by gel permeation chromatography (GPC, Tosoh Corporation, HPLC–8320 GPC, Jeonju, Korea) equipped with an RI detector. The Fourier transform infrared (FT–IR) analysis was performed to confirm the introduction of functional groups through bromination, chloromethylation, blending, and quaternization reactions. The morphology of the hydrated membranes (Br^−^, Cl^−^ forms) were characterized with the tapping mode using an atomic force microscope (AFM: nanoscope V multimode 8 AFM), installed at the Jeonju Center, Korea Basic Science Research Institute (KBSI), Jeonju, Korea. The thermal behavior of the blended membranes was evaluated using a thermal gravimetric analyzer (TGA, Q 50).

The hydroxide conductivity (σ) was measured using a four-electrode conductivity test bench (SciTech Korea, Jeonju, Korea) linked with a PGZ 301 dynamic EIS voltammeter. The ionic conductivity was calculated from the following formula:

*σ* = *L*/*RA* (1)

where *L* (cm) denotes the distance between the electrode, *R* (Ω) denotes the resistance of the membrane, and *A* (cm^2^) denotes the area of the membrane.

The activation energy (*E_a_*) of a membrane was calculated using the following equation:

*lnσ* = *lnσ_0_* − (*E_a_*/*R *× *T*) (2)

where *R* is the gas constant and *T* denotes the Kelvin temperature.

The water uptake of membranes was evaluated as follows. The sample was completely dried in an oven at 80 °C for 48 h, and the weight of the dried membrane (*W_dry_*) was measured. Afterward, the membrane was immersed in DI water at 30, 50, 70, and 90 °C for 24 h, and then the weight of the hydrated membrane (*W_wet_*) was evaluated immediately. The water uptake of the membrane was calculated using the following equation:

Water uptake (%) = ((*W_wet_* − *W_dry_*)/*W_dry_*) × 100% (3)

The swelling ratio of the membranes was determined by measuring the in-plane (∆i) and through-plane (∆t) direction before and after immersing the membrane into DI water at 30, 50, 70, and 90 °C for 24 h, respectively. The swelling ratio of the membrane was calculated by the following equation:

Swelling ratio (%) = ((*L_wet_* − *L_dry_*)/*L_dry_*) × 100% (4)

where *L_wet_* and *L_dry_* were the lengths (in-plane and through-plane directions) of fully hydrated and dried membranes, respectively.

The ion exchange capacity (IEC) of the Q-PAES/PPO-XY blended membranes were determined using the reverse titration method. The OH^−^ form membrane (10 × 50 mm) was completely dried under in a vacuum oven, and the weight of the dried membrane (M_dry_) was measured. Afterward, the membrane was immersed into 50 mL of a 0.05 M HCl solution at room temperature for 48 h. Therefore, the OH^−^ form was sufficiently replaced by the Cl^−^ form, then the membrane was removed from the HCl solution. Subsequently, the phenolphthalein indicator was added 3~4 drops to the HCl solution excluding the membrane, and was titrated with a 0.01 M NaOH solution until the solution color changed [44]. The IEC values of the Q-PAES/PPO-XY membranes were calculated using the following equation:

IEC (mmol g^−1^) = (C_HCl_ × V_HCl_ − C_NaOH_ × V_NaOH_)/M_dry_ (5)

where C_HCl_ and C_NaOH_ denote the molar concentrations of HCl and NaOH solutions, respectively, and V_HCl_ and V_NaOH_ refer to the volumes of HCl and NaOH solution reaching the equivalent point. The alkaline stability of Q-PAES/PPO-XY blended membranes was monitored by soaking them in a 2.0 M KOH solution at 50 °C for 1000 h. The blended membranes were removed from the alkaline solution, and washed repeatedly with DI water [6]. The FT-IR spectra, IEC, and TGA of the samples were observed at 200 h intervals throughout the alkaline stability test.

## 4. Discussion

### 4.1. Characterization of Br-HB-PAES and CM-PPO Polymers

The Br-HB-PAES block copolymer containing a hyperbranched structure was synthesized through three synthetic steps, as shown in Scheme 1. The HB-PAES block copolymer was prepared via direct copolymerization with HB-PAES-Cl and PAES-OH. To introduce the hyperbranched structure in the polymer main chain, the hyperbranched hydrophobic HB-PAES-Cl components were synthesized by copolymerization at a molar ratio of THPE/BPS (1.0:2.2) in the presence of K_2_CO_3_ as a basic catalyst in an anhydrous system. To optimize the conditions to form the hyperbranched structure of HB-PAES, the polymerization of HB-PAES was conducted under high temperature for a short reaction time to avoid the formation of an undesirable complex network of linear chains, as referred to in a previous report [43].

**Figure 1:**
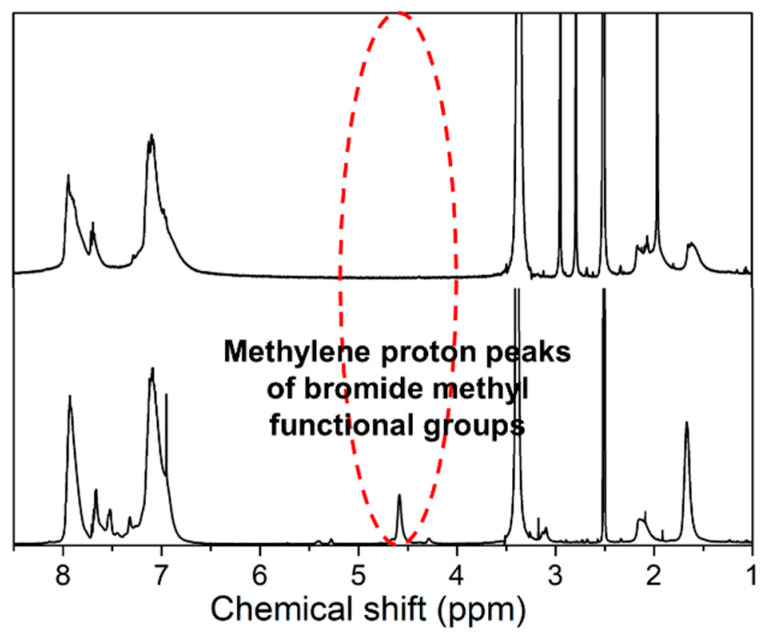
^1^H NMR spectra of the HB-PAES and Br-HB-PAES (The circle indicate the new proton peak in Br-HB-PAES).

Subsequently, the HB-PAES block copolymers were brominated using NBS as a bromination agent with BPO as a catalyst. The chemical structure of the as-prepared polymers was characterized by ^1^H NMR. As shown in Figure 1, the peaks corresponding to the main backbone were detected at 7.95–6.70 ppm and 2.15–1.46 ppm. After bromination, new proton peaks appeared at 4.47 ppm, which correspond to bromide methyl groups (–CH_2_Br). These results indicate that the brominated HB-PAES was successfully synthesized. The degree of bromination (DB) was calculated from the relative integration ratio of the bromo methyl proton groups and the unreacted benzyl methyl proton groups in the main backbone (DB: 42%) [44,45].

**Figure 2:**
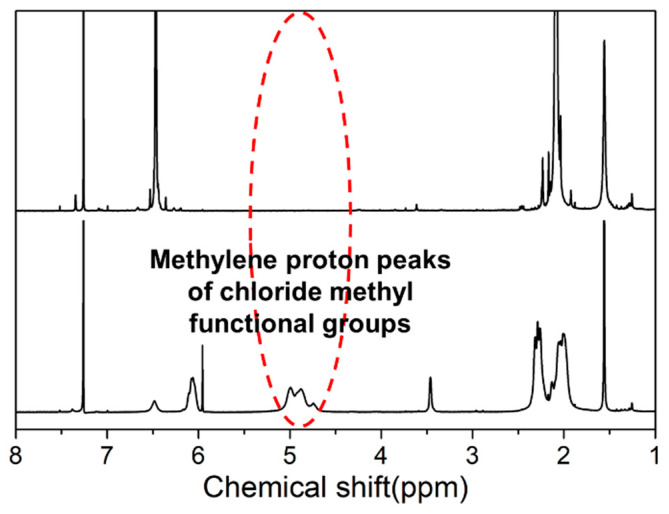
^1^H NMR spectra of the poly(phenylene oxide) (PPO) and chloromethylated poly(phenylene oxide) (CM-PPO) (The circle indicate the new proton peak in CM-PPO).

The chloromethylated PPO (CM-PPO) was prepared via the Friedel-Crafts alkylation according to a previously reported paper [8,46,47]. As shown in Scheme S1, the chloromethylation reaction of the PPO was carefully conducted to avoid the unexpected side-reactions (gelation, crosslinking, etc.) [46]. To confirm the structure and degree of chloromethylation (DC) of the as-prepared CM-PPO, ^1^H NMR was performed (Figure 2). New proton peaks appeared around 4.99–4.67 ppm, which correspond to the chloride methyl groups (–CH_2_Cl). The DC was calculated from the relative integration ratio of the chloride methyl proton peaks and unreacted benzyl proton peaks in the main backbone (DC: 51%).

### 4.2. Fabrication and Charaterization of Blended Membranes

**Figure 3:**
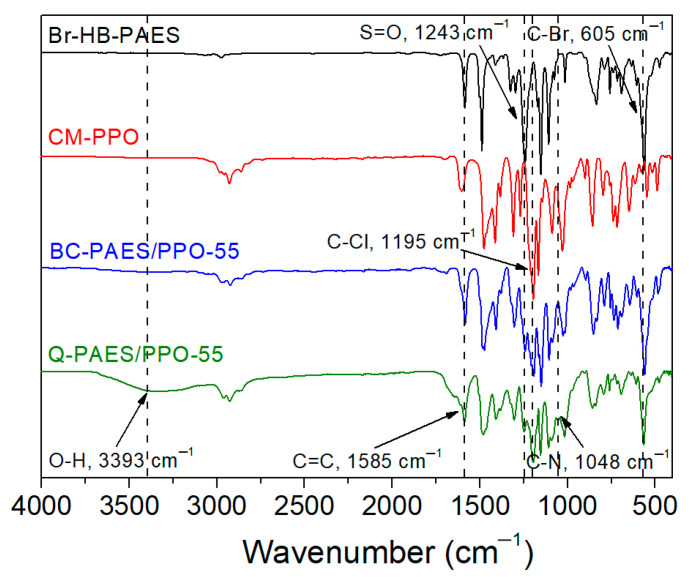
FT-IR spectra of Br-HB-PAES, CM-PPO, BC-PAES/PPO-55 blended membrane, and Q-PAES/PPO-55 blended membrane.

As listed in Table 1, a series of BC-PAES/PPO-XY blended membranes were prepared by controlling the mixing weight ratio of Br-HB-PAES to Cl-PPO. The fabricated membranes were macroscopically homogeneous and were uniformly mixed to form transparent membranes. The chemical structures of Br-HB-PAES, CM-PPO, BC-PAES/PPO-55, and Q-PAES/PPO-55 were confirmed by FT-IR, as illustrated in Figure 3. The IR bands of the BC-PAES/PPO-55 blended membrane showed two major peaks at 605 and 1190 cm^−1^, which are assigned to C-Br and C-Cl stretching vibrations, respectively. In addition, a vibration corresponding to sulfone (S=O) appeared at 1243 cm^−1^. The absorption peak at 1585 cm^−1^ is ascribed to the stretching vibration of C=C in aromatic benzene rings, indicating that the BC-PAES/PPO-55 blended membrane was successfully blended. The IR spectrum of the Q-PAES/PPO-55 blended membrane includes a peak at 3393 cm^−1^ from the stretching vibration of O-H groups and a peak around 1048 cm^−1^ from the stretching vibration of C-N bands [48,49]. The results show that the blended membranes were successfully fabricated and the quaternization reaction was carried out well.

The solubility of the obtained BC-PAES/PPO-XY blended membranes was measured in various organic solvents at 40 °C. As listed in Table 2, the BC-PAES/PPO-XY blended membranes dissolved easily in polar aprotic solvents such as dimethyl sulfoxide (DMSO), *N*,*N*-dimethylformamide (DMF), tetrahydrofuran (THF), trichloroethylene (TCE), N-methyl-2-pyrrolidone (NMP), and dichloromethane (MC).

### 4.3. Morphology of Q-PAES/PPO-XY Blended Membranes

**Figure 4:**
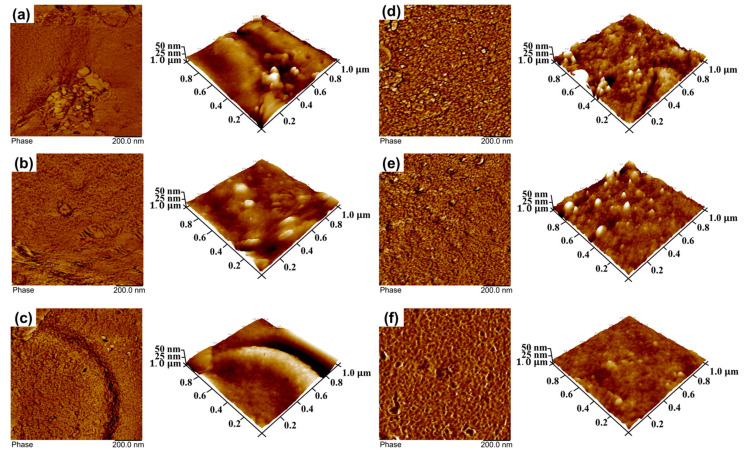
Atomic force microscope (AFM) phase images and three-dimensional (3D) images of (**a**) the pristine Q-HB-PAES membrane, (**b**) Q-PAES/PPO-73, (**c**) Q-PAES/PPO-64, (**d**) Q-PAES/PPO-55, (**e**) Q-PAES/PPO-46, and (**f**) Q-PAES/PPO-37 blended membranes.

The micro phase-separation in morphology helps improve the dimensional stability and anion conductivity of AEMs. Therefore, the morphology of AEMs with different blending ratios of Q-HB-PAES and Q-PPO was investigated by the AFM analysis and the results are illustrated in Figure 4. As shown, the dark regions corresponding to hydrophilic areas and the bright regions corresponding to hydrophobic areas were observed, demonstrating that the morphology of all AEMs have the distinct micro phase-separation [10]. It proved that the Q-PAES/PPO-XY blended membranes formed a well-interconnected ion cluster with increasing the Q-PPO block ratio in the blending membrane due to the aggregation of hydrophilic regions resulting from the electrostatic interaction (N^+^/OH^−^) and H-bonding interaction (OH^−^/H_2_O) between Q-HB-PAES and Q-PPO [50,51,52]. Therefore, the introduction of the hyperbranching network structure in the polymer matrix would promote the enhanced ion network between the ionic groups, which provides a well-connected ion transportation.

### 4.4. Ionic Exchange Capacity, Water Uptake, and Swelling Ratio

**Figure 5:**
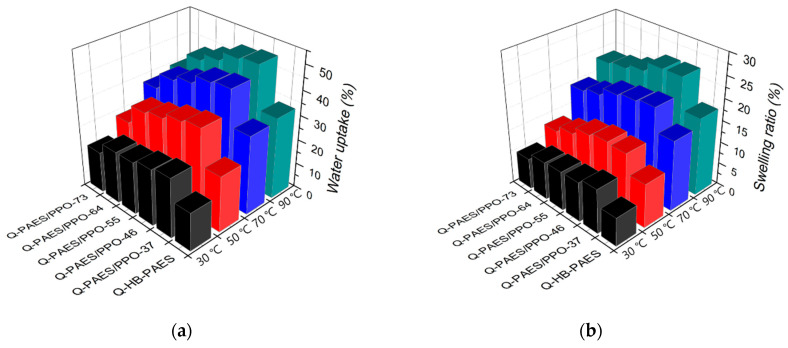
(**a**) Water uptake and (**b**) swelling ratio of the pristine Q-HB-PAES membrane and the Q-PAES/PPO-XY blended membranes.

The water uptake and swelling ratio are affected by the IEC values. In general, an increase in the water uptake leads to the increasing capacity to transport OH^−^ ions in the membrane, improving the IEC values [6,36]. However, the excessive water uptake by the membrane attenuates a dimensional stability [53,54]. The IEC, water uptake, and swelling ratio of the as-prepared membranes were measured at 30, 50, 70, and 90 °C, as presented in Figure 5 and Table 3. The IEC values of the pristine Q-HB-PAES membrane and the Q-PAES/PPO-XY blended membranes were 1.27 and 1.32–1.96 mmol g^−1^, respectively. In addition, the water uptake and swelling ratio of the Q-PAES/PPO-XY blended membranes exhibited 34.6–50.3% and 20.9–25.0% at 90 °C, respectively. The Q-PAES/PPO-XY blended membranes showed a suitable water uptake, low swelling ratio, and IEC value due to the bulky hyperbranched structure and the relatively strong electrostatic interactions and H-bonding between the blending materials. These membranes can hold more water within the polymer matrix [39,51,52,55,56], compared to the Q-HB-PAES membrane. Furthermore, the IEC value of the Q-PAES/PPO-55 blended membranes, with the most miscible blending weight ratio, was 1.84 mmol g^−1^.

### 4.5. Hydroxide Conductivity and Arrhenius Plots of the Q-PAES/PPO-XY Blended Membranes

**Figure 6:**
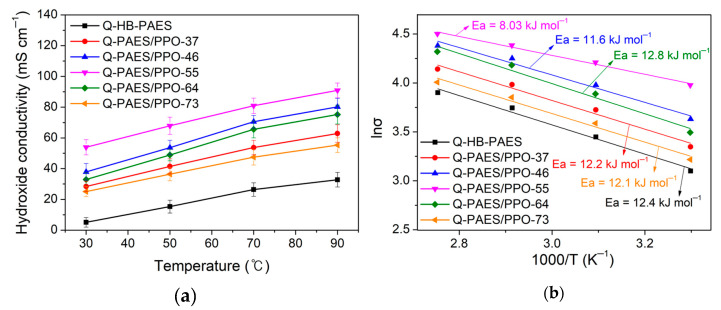
Temperature dependence (30, 50, 70, and 90 °C, 100% RH) of (**a**) hydroxide conductivity and (**b**) Arrhenius plots of the pristine Q-HB-PAES membrane and the Q-PAES/PPO-XY blended membranes.

**Figure 7:**
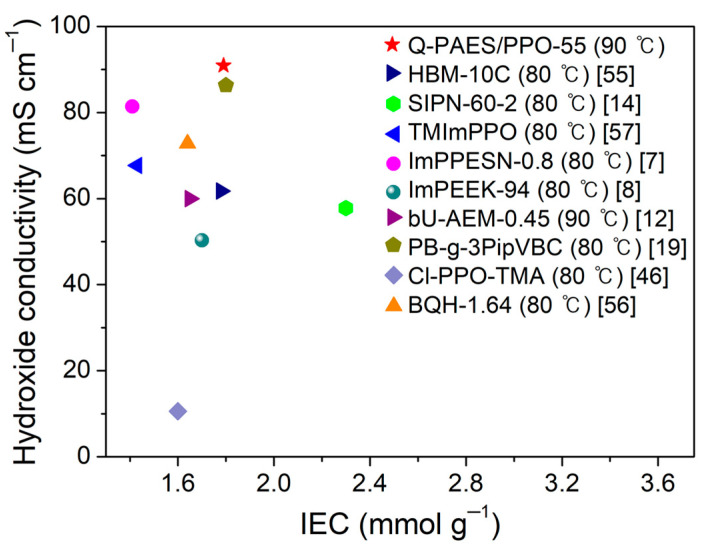
Comparison of hydroxide conductivity and ion exchange capacity (IEC) values of the recently reported anion exchange membranes (AEMs).

As listed in Table 4, the hydroxide conductivity of the AEMs, an important parameter, that plays a significant role in fuel cell performance, steadily increased with the temperature due to the enhanced water mobility [28], and was influenced by IEC. High IEC values facilitate the ion transport, which improves the water uptake [29]. The ionic conductivity of the membrane must exceed 10 mS cm^−1^ at RT for the AEM to be applied to fuel cells [28,38]. Figure 6a shows the four-probe hydroxide conductivities of the pristine Q-HB-PAES membrane, and the Q-PAES/PPO-XY blended membranes. The results indicate an increased miscibility between Q-HB-PAES and Q-PPO mediated by the electrostatic interaction and H-bonding interaction, as well as an increased hydroxide conductivity from 25.1–53.9 mS cm^−1^ at 30 °C and from 55.3–90.9 mS cm^−1^ at 90 °C. In particular, the Q-PAES/PPO-55 blended membranes exhibited the highest ionic conductivity value. Due to fact that the balance of two polymers in the blended membranes via electrostatic interactions and H-bonding interactions are increased, it is believed that the Q-PAES/PPO-55 membrane has a more well-defined hydrophilic/hydrophobic microphase separation to form wide ion conducting channels than the other Q-PAES/PPO-XY membranes (37, 46, 64, and 73) [35,51]. As shown in the Arrhenius plots (Figure 6b), the achieved activation energy (E_a_) of the as-prepared pristine membrane and blending membranes ranged from 8.03 to 12.80 kJ mol g^−1^, indicating that the ion transportation mechanism followed the vehicle mechanism [30]. Moreover, the hydroxide conductivity comparison graph with similar IEC values of the recently reported AEMs is shown in Figure 7 [7,8,12,14,19,30,46,55,56,57].

### 4.6. Thermal Properties

**Figure 8:**
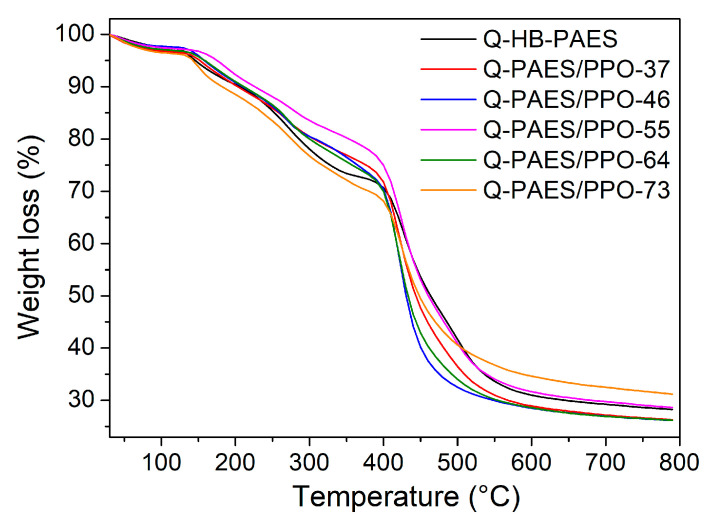
TGA curves of the Q-PAES/PPO-XY blended membranes (temperature range: 30–800 °C, heating ramp rate: 10 °C min^−1^).

The thermal properties of AEMs are important for the practical application of AEMFCs, which are operated between 80–100 °C. As shown in Figure 8, the thermal degradation of the Q-PAES/PPO-XY blended membranes was investigated by TGA at a temperature rising rate of 10 °C min^−1^ in nitrogen atmosphere, which showed three weight loss stages. The initial stage around 135–180 °C is attributed to the volatilization of water absorbed in the membranes. The second stage of weight loss in the range of 180–400 °C corresponds to the decomposition of quaternary ammonium groups. The third stage of weight loss (above 400 °C) is assigned to the decomposition of the polymer main chains [42,58].

### 4.7. Alkaline Stability

**Figure 9:**
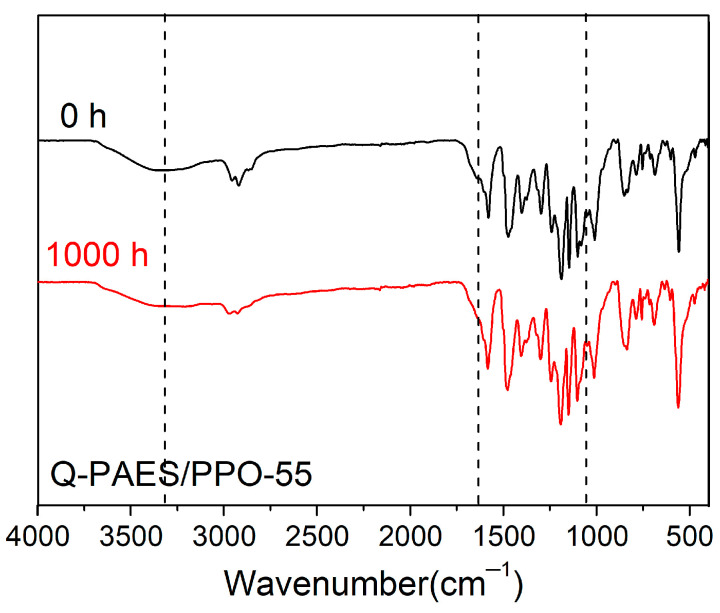
FT-IR spectra of Q-PAES/PPO-55 blended membranes before and after the alkaline stability test (immersed in a 2.0 M KOH aqueous solution at 50 °C for 1000 h).

**Figure 10:**
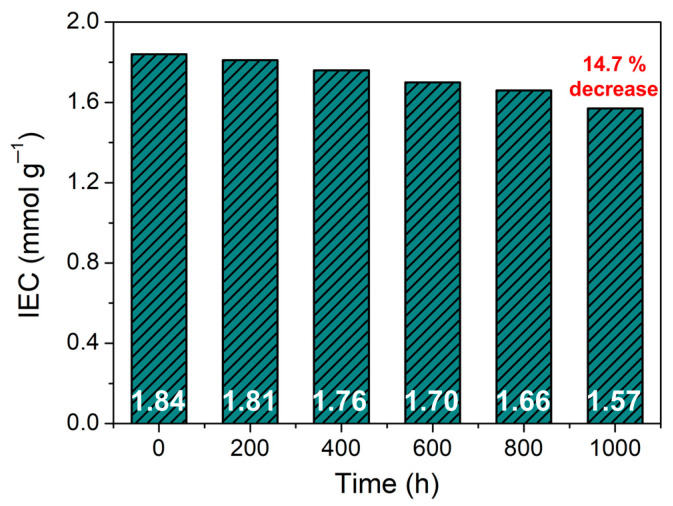
IEC of Q-PAES/PPO-55 blended membranes before and after the alkaline stability test (immersed in a 2.0 M KOH aqueous solution at 50 °C for 1000 h).

The alkaline stability of AEMs is a critical parameter, AEMs must operate in high pH environments in FC applications [59,60,61,62]. In general, the quaternary ammonium groups of the AEMs disintegrate in a harsh alkaline condition as the result of the OH^−^ attack via Hofmann elimination and direct nucleophilic substitution [47,63]. In the present work, the Q-PAES/PPO-55 blended membranes achieved the highest electrochemical performance and dimensional stability. Its chemical stability was tested by immersion in a 2.0 M KOH aqueous solution at 50 °C for 1000 h, followed by the evaluation of its FT-IR spectra and IEC. As shown in Figure 9, the transition of the chemical structure of the AEMs resulting from the alkaline stability test was characterized via the FT-IR spectra. A small change around 3393 cm^−1^ was observed, corresponding to the O-H stretching vibration, and there was a slight change of the peak at 1048 cm^−1^ corresponding to the C-N stretching vibration. However, there was no significant visible change in the overall spectrum. Moreover, at 200 h intervals during the prolonged alkaline treatment, the Q-PAES/PPO-55 blended membranes were tested by the IEC titration method. It turns out that the IEC values remain at 85.3% during immersion in a high pH environment for the alkaline stability test, as shown in Figure 10. The assembly of the bulky hyperbranched structure and the linear structure protects the backbone and quaternary ammonium groups from the OH^−^ attack through the steric hindrance effect. The result showed that chemically stable Q-PAES/PPO-XY blended membranes were obtained [31,33,55].

## 5. Conclusions

In summary, a series of Q-PAES/PPO-XY blended membranes were prepared by simply blending hyperbranched brominated PAES and linear chloromethylated PPO different weight ratios (30, 40, 50, 60, and 70 wt%) to improve the hydroxide conductivity and dimensional stability of AEMs. The Q-PAES/PPO-XY membranes form electrostatic interaction and hydrogen bonding networks between the quaternized functional groups introduced in the HB-PAES and PPO, and the blended membranes showed excellent dimensional/chemical properties, depending on the mixing ratio of the polymers, compared to the pristine Q-HB-PAES membrane. The dense and concentrated ammonium groups promoted the nanophase separation of the AEM and showed excellent electrochemical properties despite the low IEC. Among the blended membranes, the Q-PAES/PPO-55 membrane achieved the highest hydroxide ion conductivity (90.9 mS cm^−1^ at 90 °C) and showed a reasonable alkaline stability under high pH conditions. Therefore, the developed blended membranes are a promising candidate for AEM fuel cell applications.

## Figures and Tables

**Table 1:** Different weight ratios of BC-PAES/PPO-XY blended membranes.

Samples	Br-HB-PAES (%)	CM-PPO (%)
BC-PAES/PPO-37	30	70
BC-PAES/PPO-46	40	60
BC-PAES/PPO-55	50	50
BC-PAES/PPO-64	60	40
BC-PAES/PPO-73	70	30

**Table 2:** The solubility of BC-PAES/PPO-XY blending membranes.

Membrane	DMSO	DMF	THF	TCE	NMP	MC	Chloroform	Acetone	Methanol
BC-PAES/PPO-37	++	++	++	++	++	++	++	-	--
BC-PAES/PPO-46	++	++	++	++	++	++	++	-	--
BC-PAES/PPO-55	++	++	++	++	++	++	++	-	--
BC-PAES/PPO-64	++	+	++	++	++	++	+	--	--
BC-PAES/PPO-73	++	++	++	++	++	++	+	--	--

++: Highly soluble; +: Soluble; -: Partially soluble; --: Insoluble.

**Table 3:** Ionic exchange capacity, water uptake, and swelling ratio of the pristine Q-HB-PAES membrane and the Q-PAES/PPO-XY blended membranes.

Membranes	Water Uptake (%)				Swelling Ratio (%)				IEC (mmol g^−1^)
					∆i		Δt		
	30 °C	50 °C	70 °C	90 °C	30 °C	90 °C	30 °C	90 °C	
Q-HB-PAES	15.5	22.7	32.0	33.2	6.5	17.9	7.1	18.2	1.24 ± 0.03
Q-PAES/PPO-37	24.3	37.1	46.1	50.3	10.2	25.0	12.1	27.1	1.97 ± 0.04
Q-PAES/PPO-46	23.0	35.5	45.0	49.2	9.1	24.9	11.0	28.0	1.90 ± 0.03
Q-PAES/PPO-55	21.3	32.9	41.4	45.4	9.0	22.7	10.7	25.7	1.79 ± 0.05
Q-PAES/PPO-64	21.2	31.3	39.1	42.3	8.7	21.5	9.2	22.9	1.32 ± 0.04
Q-PAES/PPO-73	16.7	23.5	32.5	34.7	7.1	20.9	8.6	21.4	1.27 ± 0.05

**Table 4:** The hydroxide conductivity of the Q-PAES/PPO-XY blended membranes according to the blending weight ratio by temperature.

Membranes	Hydroxide Conductivity (mS cm^−1^)			
	30 °C	50 °C	70 °C	90 °C
Q-HB-PAES	5.1	15.3	26.4	32.8
Q-PAES/PPO-37	28.4	41.5	53.7	62.9
Q-PAES/PPO-46	37.9	53.7	70.5	80.2
Q-PAES/PPO-55	53.9	67.9	80.9	90.9
Q-PAES/PPO-64	32.9	48.9	65.5	75.2
Q-PAES/PPO-73	25.1	36.4	47.4	55.3

## Supplementary Materials

The following are available online at https://www.mdpi.com/2073-4360/12/12/3011/s1, Figure S1: ^1^H NMR spectra (a) hydrophobic oligomer (HB-PAES-Cl), (b) hydrophilic precursor (PAES-OH), and (c) block copolymer (HB-PAES), Scheme S1: Synthesis process of chloromethylated poly(phenylene oxide) (CM-PPO), Figure S2: In-plane swelling ratios and through-plane swelling ratios of (1) Q-HB-PAES pristine membrane, (2) Q-PAES/PPO-37, (3) Q-PAES/PPO-46, (4) Q-PAES/PPO-55, (5) Q-PAES/PPO-64, and (6) Q-PAES/PPO-73 blended membranes.
