# Supplementary material for: Enhanced Hydroxide Conductivity and Dimensional Stability with Blended Membranes Containing Hyperbranched PAES/Linear PPO as Anion Exchange Membranes
Source: Polymers (Basel). 2020 Dec 16;12(12):3011. doi: 10.3390/polym12123011 (PMC7766666; doi:10.3390/polym12123011)
Supplement: Supplementary file 1 [file polymers-12-03011-s001.pdf]

polymers

MDPI

Supplementary materials

Enhanced Hydroxide Conductivity and Dimensional Stability with Blended Membranes Containing Hyperbranched PAES/Linear PPO as Anion Exchange Membrane

Sang Hee Kim[1], Kyu Ha Lee[2], Ji Young Chu[2], Ae Rhan Kim[2], Dong Jin Yoo[1,2,*]

[1] Department of Energy Storage/Conversion Engineering of Graduate School, Hydrogen and Fuel Cell Research Center, and Education Center for Whole Life Cycle R&D of Fuel Cell Systems, Jeonbuk; tkdgml3257@gmail.com

[2] Department of Life Sciences, Jeonbuk National University, Jeonju 54896, Republic of Korea ;

***corresponding author: djyoo@jbnu.ac.kr**

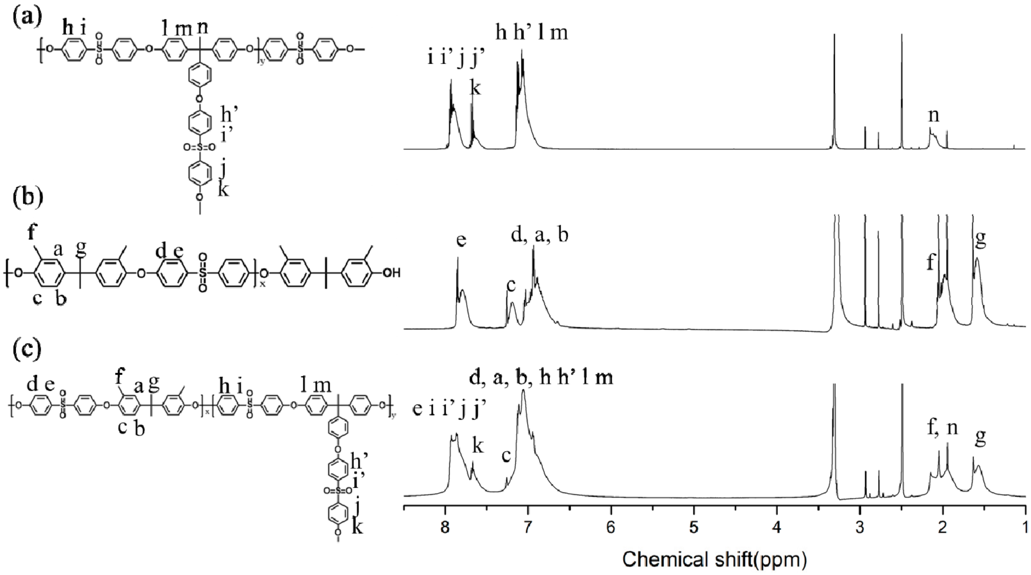

Figure S1. ^{1}H NMR spectra (a) hydrophobic oligomer (HB-PAES-Cl), (b) hydrophilic precursor (PAES-OH), and (c) block copolymer (HB-PAES).

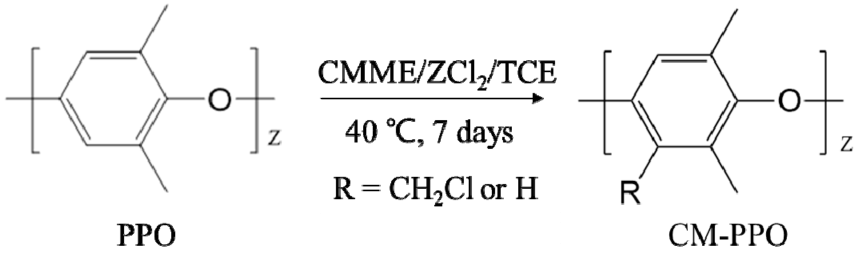

Scheme S1. Synthesis process of chloromethylated poly(phenylene oxide) (CM-PPO).

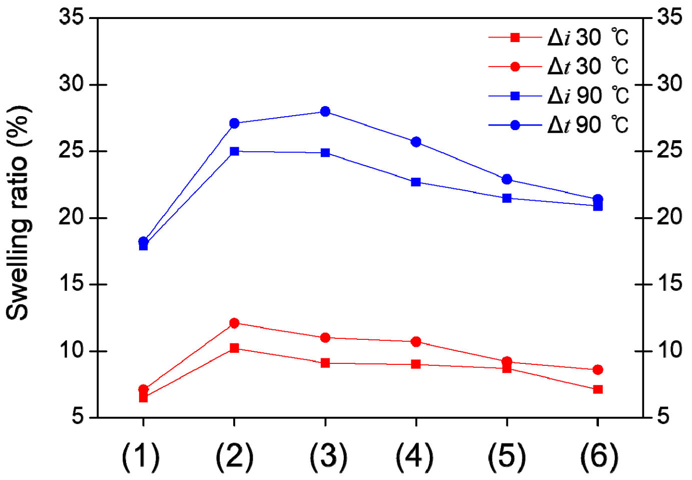

Figure S2. In-plane swelling ratios and through-plane swelling ratios of (1) Q-HB-PAES pristine membrane, (2) Q-PAES/PPO-37, (3) Q-PAES/PPO-46, (4) Q-PAES/PPO-55, (5) Q-PAES/PPO-64, and (6) Q-PAES/PPO-73 blended membranes.

© 2020 by the authors. Submitted for possible open access publication under the terms and conditions of the Creative Commons Attribution (CC BY) license (http://creativecommons.org/licenses/by/4.0/).
